# Paradigm shift in aerosol chemical composition over regions downwind of China

**DOI:** 10.1038/s41598-020-63592-6

**Published:** 2020-04-15

**Authors:** Itsushi Uno, Zhe Wang, Syuichi Itahashi, Keiya Yumimoto, Yuki Yamamura, Ayako Yoshino, Akinori Takami, Masamitsu Hayasaki, Byung-Gon Kim

**Affiliations:** 1 Research Institute for Applied Mechanics, Kyushu University, Fukuoka, Japan; 2 State Key Laboratory of Atmospheric Boundary Layer Physics and Atmospheric Chemistry (LAPC), Institute of Atmospheric Physics (IAP), Chinese Academy of Sciences (CAS), Beijing, China; 3 Central Research Institute of Electric Power Industry, Abiko Chiba, Japan; 4 Fukuoka Institute of Health and Environmental Science, Dazaifu Fukuoka, Japan; 5 National Institute for Environmental Studies, Tsukuba Ibaraki, Japan; 6 Japan Automobile Research Institute, Tsukuba Ibaraki, Japan; 7 Gangneung-Wonju National University, Gangneung, Korea

**Keywords:** Environmental chemistry, Atmospheric chemistry

## Abstract

A rapid decrease in PM_2.5_ concentrations in China has been observed in response to the enactment of strong emission control policies. From 2012 to 2017, total emissions of SO_2_ and NO_x_ from China decreased by approximately 63% and 24%, respectively. Simultaneously, decreases in the PM_2.5_ concentration in Japan have been observed since 2014, and the proportion of stations that satisfy the PM_2.5_ environmental standard (daily, 35 µg/m^3^; annual average, 15 µg/m^3^) increased from 37.8% in fiscal year (FY) 2014 (April 2014 to March 2015) to 89.9% in FY 2017. However, the quantitative relationship between the PM_2.5_ improvement in China and the PM_2.5_ concentration in downwind regions is not well understood. Here, we (1) quantitatively evaluate the impacts of Chinese environmental improvements on downwind areas using source/receptor analysis with a chemical transport model, and (2) show that these rapid emissions reductions improved PM_2.5_ concentrations both in China and its downwind regions, but the difference between SO_2_ and NO_x_ reduction rates led to greater production of nitrates (e.g., NH_4_NO_3_) due to a chemical imbalance in the ammonia–nitric acid–sulfuric acid–water system. Observations from a clean remote island in western Japan and numerical modeling confirmed this paradigm shift.

## Introduction

The long-range trans-boundary transport behavior of pollutants in East Asia is an important environmental issue due to frequent outflows of heavy pollution. Among pollutants, PM_2.5_ (particulate matter less than 2.5 µm in diameter) poses serious human health risks, including lung cancer, respiratory disease, and asthma, particularly over China and its downwind regions^1–5^. Serious PM_2.5_ pollution has been observed in the northern China region since the early 2010s. To reduce this pollution, China has implemented active clean air policies in recent years (e.g., the Action Plan for Prevention and Control of Air Pollution, enacted in September 2013). These plans include the phasing out of outdated industrial capacity, small high-emission factories, and small coal-fired industrial boilers, as well as the strengthening of emission standards for power plants, industries, and vehicles, and the replacement of residential coal use with electricity and natural gas^6,7^. These strong emission-reduction policies in China have led to a successful reduction in PM_2.5_ concentration (e.g., PM_2.5_ concentrations decreased from 102 µg/m^3^ in 2013 to 43 µg/m^3^ in 2019, as observed at the U.S. embassy in Beijing). Rapid reductions in SO_2_ and NO_x_ emissions were also confirmed using environmental satellite data^8^ and bottom-up emissions inventory studies^1^. MODIS AOD (aerosol optical depth) data revealed that there has been a consistent trend of year-to-year decreases in China and its downwind regions. The chemical composition of PM_2.5_ also changed significantly over China, with especially large decreases in levels of organic matter, mineral components, and sulfate aerosols^9^. Studies have suggested that emission control has a dominant effect on PM_2.5_ reduction compared to inter-annual meteorological variation^10–12^. Further research using bottom-up emission inventory and numerical models revealed that different emission control measures contribute to reductions in PM_2.5_^6,13^. Meanwhile, recent studies have reported significant health benefits resulting from PM_2.5_ improvements in China^14,15^.

Focusing on regions downwind of China, the proportion of monitoring stations meeting the Japanese environmental standard for PM_2.5_ (defined as the achievement ratio) increased rapidly from 37.8% in fiscal year (FY) 2014 to 89.9% in FY 2017 (Ministry of Environment, Japan)^16^. During this period, Japanese pollutant emissions exhibited a slight decreasing trend^17^. Improvements in the PM_2.5_ achievement ratio result from complex interactions between Japanese and Chinese emission-reduction measures, and it is not yet clear which factor has a greater contribution. This lack of clarity arises because most PM_2.5_ studies are focused on an individual country, and quantitative evaluations or correlation analyses of the impacts of environmental improvement on downwind regions (beyond national borders) have rarely been published^18–21^. Studying the relationship between decreased PM_2.5_ concentrations in China and its downwind regions and clarifying quantitative source/receptor (S/R) relationships are essential for establishing better environmental policies.

Remote island observations of aerosol chemical compositions off the west coast of Japan (eastern edge of the East China Sea) indicate that sulfate concentrations have decreased significantly, consistent with the decrease in SO_2_ emissions in China. Although NO_x_ emissions also decreased, the observed nitrate concentration increased continuously in recent years^22^. This increase in the nitrate concentration could lead to excess N input to the oceans surrounding East Asia, which may have impacts on the marine ecosystem^23^. In this paper, we support this observational finding with chemical transport model (CTM) sensitivity experiments, in which SO_2_ and NO_x_ emissions are reduced at different rates to confirm the observed trend. These experiments showed that the observed nitrate increase can be explained by changes in the ammonia–nitric acid–sulfuric acid–water system balance due to the greater rate of decrease in SO_2_ emissions compared to that in NO_x_ emissions.

## Results

We analyzed hourly surface-level PM_2.5_ observational data from Japan and China and calculated the annual average PM_2.5_ concentration for each region. For Korea, we used annual average values from the Air Korea website. The observational data used in this study are described in the Methods section.

Satellite observations of NO_2_ and SO_2_ from the Ozone Monitoring Instrument (OMI) were also used for the analysis of emission trends between 2005 and 2019. Gridded (0.25 × 0.25 degree) Level 3 data from NASA were used for this study and annually-averaged data were used to examine year-to-year trends over China, Korea, and Japan (NASA OMI website)^24^.

**Figure 1:**
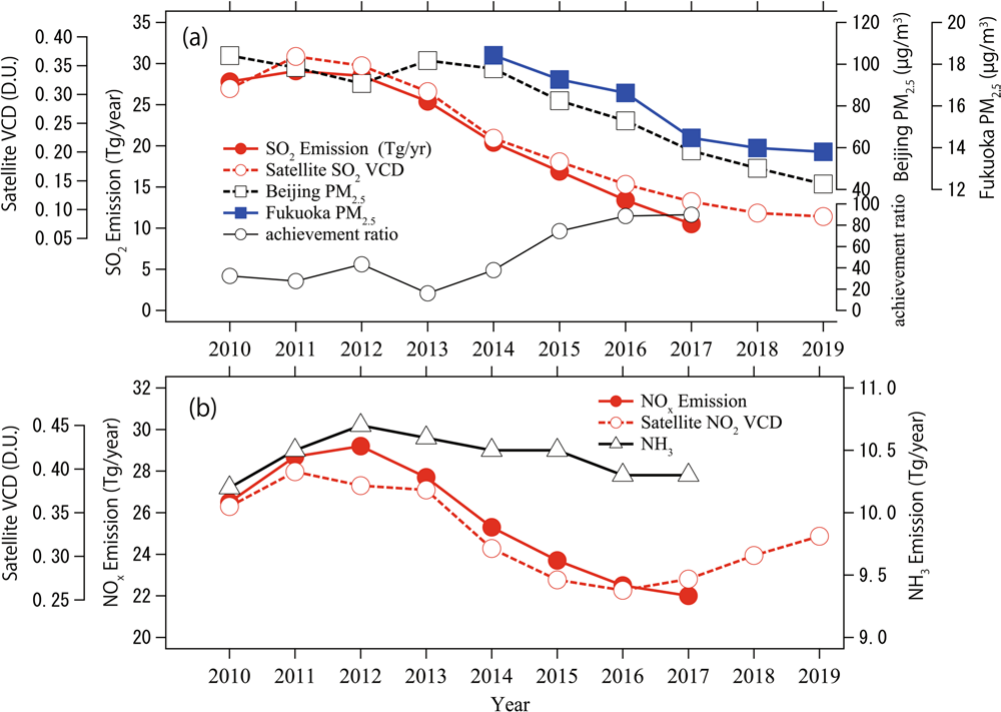
(**a**) Year-to-year trends of annually averaged PM_2.5_ concentrations at Beijing and Fukuoka, SO_2_ emissions from China^1^, vertical column densities of SO_2_ over central eastern China (CEC) as calculated from OMI satellite data^24^, and PM_2.5_ achievement ratios for Japan. (**b**) Year-to-year trends of NO_x_ and NH_3_ emissions from China^1^ and vertical column densities of NO_2_ over CEC as calculated from OMI satellite data^24^.

Figure 1 shows the annual average PM_2.5_ concentrations over Fukuoka, Japan and Beijing (at the U.S. Embassy), China (Supplementary Figure S1 shows the locations of the observation sites). This figure includes estimated SO_2_, NO_x_, and NH_3_ emissions over China^1^, and tropospheric vertical column densities (VCDs) of SO_2_ and NO_2_ over central eastern China (CEC) (OMI satellite data from 2011 to 2019)^24^. This figure also shows the PM_2.5_ achievement ratio for Japan. Similar plots for the average of 74 cities in China and Korea (including two background sites), and several remote Japanese sites are provided in Supplementary Figure S2. Supplementary Figure S3 shows the annual average VCDs of SO_2_ and NO_2_ levels in East Asian regions between 2011 and 2019 based on satellite retrieval data. Supplementary Figure S4 shows the year-to-year average trends in three regions (CEC, Korea, and Japan) for SO_2_ and NO_2_ levels based on satellite observations.

The PM_2.5_ trends in Beijing and the average trend of 74 Chinese cities were very strongly correlated (Supplementary Figure S2(a)), although the PM_2.5_ concentration in Beijing was 40% higher than the average concentration of 74 cities in 2013 (this difference became negligible in 2018, as the rate of decrease in Beijing was greater). This trend was quite consistent with that in Japan (Supplementary Figure S2(c)). The correlation coefficient (R) between Beijing and Fukuoka was 0.98. The average PM_2.5_ trend for Korea (Supplementary Figure S2(b)) also showed a generally decreasing trend but with a slight difference from those in China and Japan up to 2017.

Bottom-up inventory results and satellite data also exhibit good correlations (Fig. 1). OMI SO_2_ observations (Supplementary Figure S3) indicated that a rapid decrease in SO_2_ was achieved over the CEC area, and the color representing SO_2_ in the image has been nearly absent since 2017 (using the same color scale). We found that the SO_2_ VCDs in Korea and Japan exhibited small decreases or constant levels after 2010 (see Supplementary Figure S4), whereas NO_2_ VCDs in Korea and Japan remained almost constant and increased slightly, respectively, since 2017. NO_2_ VCDs exhibited an increasing trend over CEC after 2016.

**Figure 2:**
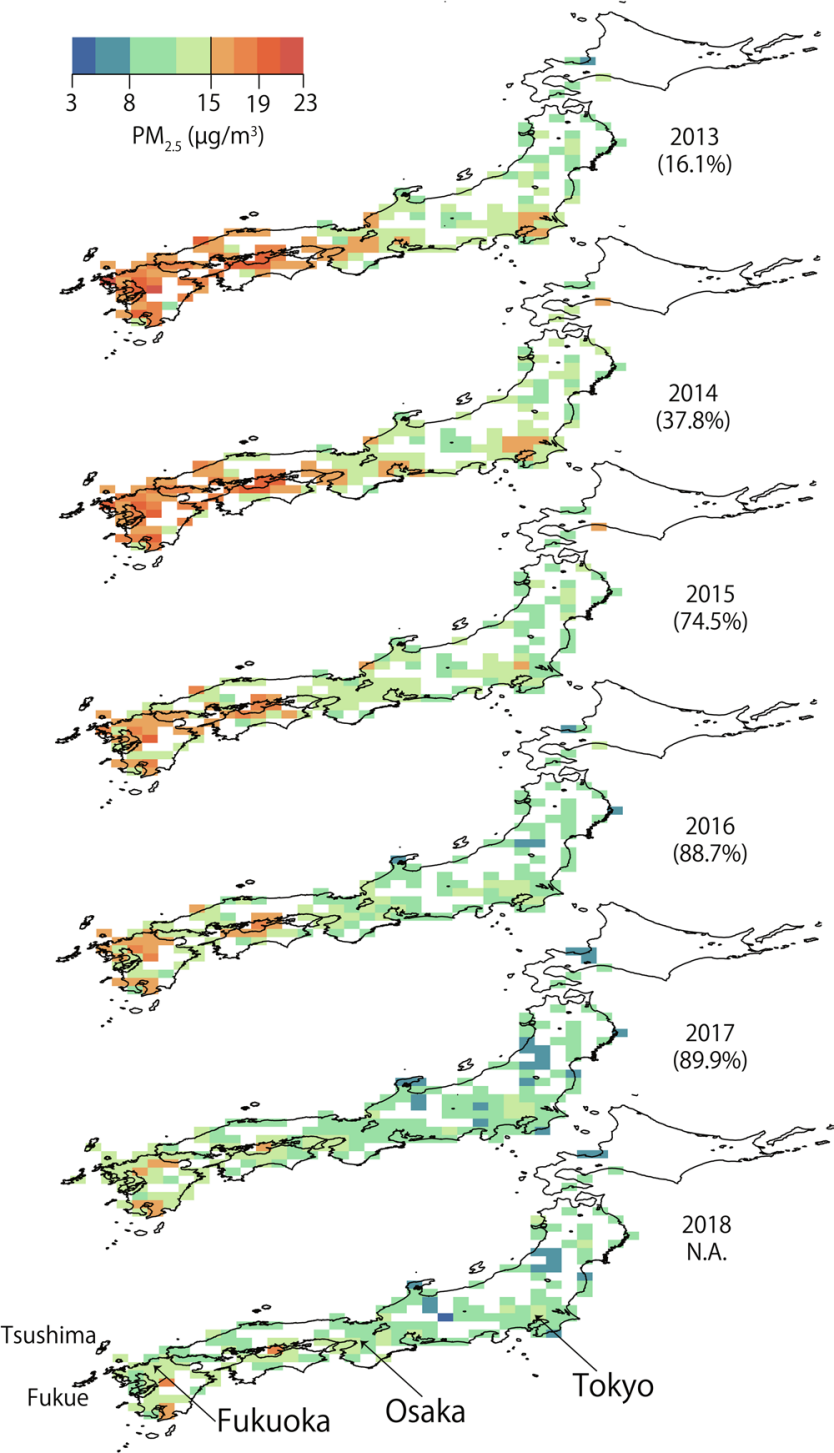
Horizontal distribution of annually averaged PM_2.5_ concentrations over Japan between 2013 and 2018. Numbers in parentheses indicate PM_2.5_ achievement ratios for Japan.

Figure 2 shows the horizontal distribution of annually-averaged PM_2.5_ in Japan from 2013 to 2018. The numbers in parentheses indicate the achievement ratios. PM_2.5_ values were higher in the western part of Japan compared to the Tokyo metropolitan area. This pattern of high values in the west and low values in the east (i.e., a strong west-east gradient) indicates that the PM_2.5_ concentration was strongly influenced by trans-boundary pollution from the west of Japan. From the year-to-year changes in PM_2.5_, we found that the PM_2.5_ concentration over large areas of Japan decreased rapidly from 2014 to 2015, with the achievement ratio increasing from 37.8% to 74.5% within one year. It is important to note that there was a high rate of decrease in PM_2.5_ from 2014 to 2017, but the rate slowed down after 2017. This is because the recent decrease in the trans-boundary fraction is significant, and the improvement was dramatic and rapid across the rural/remote sites impacted by the trans-boundary fractions. The domestic emissions from large urban and industrial areas contribute greatly to the PM_2.5_ concentration, at levels near or sometimes exceeding the criteria. In 2018, a few PM_2.5_ hotspots could be observed in a very limited area with strong local effects from volcanoes and industrial emissions.

**Figure 3:**
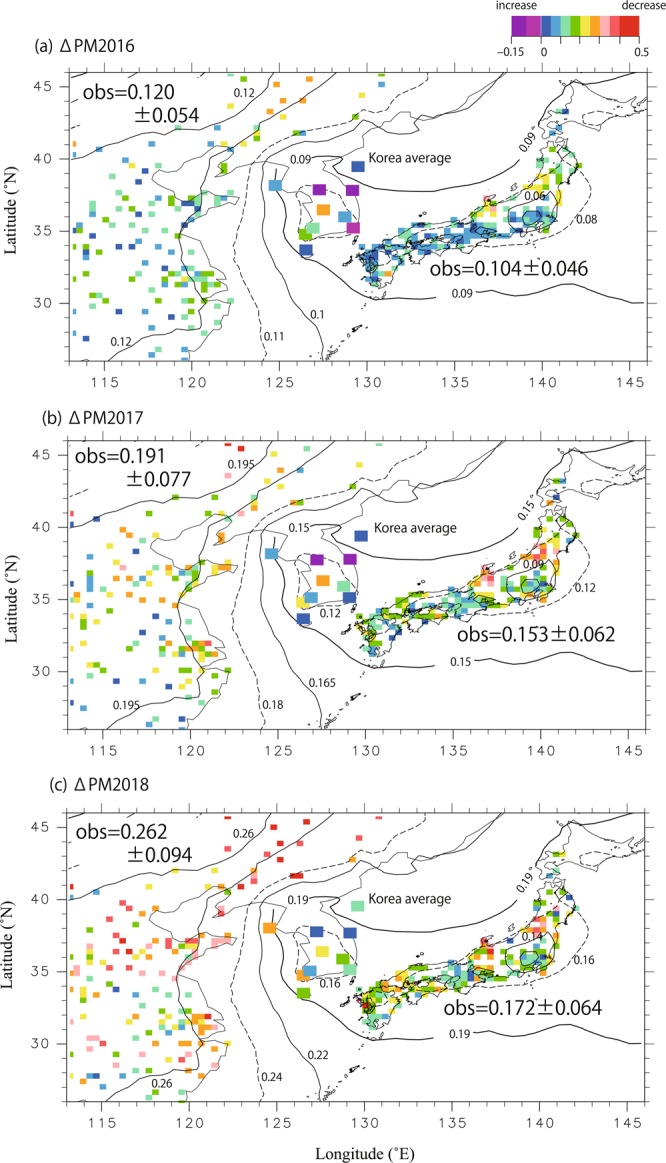
(**a**) Horizontal distribution of the rates of decrease in annually averaged PM_2.5_ concentrations in 2016 compared with 2015 (see Eq. 1 for definitions). (**b**) Same as (**a**) but for 2017. (**c**) Same as (**a**) but for 2018. Note that the colored squares indicate observed rates of decrease in PM_2.5_ concentrations, and contour lines represent rates of decrease over East Asia in response to different rates of decrease in PM_2.5_ concentrations over China, as estimated from the simulated source-receptor (S/R) relationship.

Figure 3 is similar to Fig. 2 but covers an extended region including Eastern China and Korea. Different colors in the figure represent different rates of decrease in observed PM_2.5_ levels, defined as:

1 $${\Delta {\rm{PM}}}_{{\rm{yyyy}}}=({{\rm{PM}}}_{2.5\_2015}-{{\rm{PM}}}_{2.5\_{\rm{yyyy}}}){/{\rm{PM}}}_{2.5\_2015},$$

where yyyy indicates a year. The rate was scaled based on the concentration in 2015. Negative values of ΔPM_yyyy_ indicate that PM_2.5_ increased relative to 2015, as seen at some Korean sites. The annual average ΔPM_yyyy_ values over Eastern China (regionally averaged over 115°–123° E, 28°–43° N) from 2016 to 2018 were 0.120 ± 0.054, 0.191 ± 0.077, and 0.262 ± 0.094, respectively. This result indicates that the observed average PM_2.5_ concentration over Eastern China decreased by approximately 7% annually between 2016 and 2018.

The detailed changes over Japan were discussed in the context of Fig. 2. Figure 3 shows observed annual average rates of decrease (regionally averaged over 130°–142° E, 33°–37° N) of 0.104 ± 0.046, 0.151 ± 0.062, and 0.172 ± 0.064 since 2015.  ΔPM_yyyy_ values for both the Korean average and individual stations are shown. A complicated variation in ΔPM_yyyy_ was observed, with some stations exhibiting positive changes or different variation patterns between years, except at the upwind background stations in Baengyeongdo and Jeju, which will be discussed later.

We used the 3-D Goddard Earth Observing System chemical transport model (GEOS-Chem)^25^ for emission sensitivity analysis, including that of the S/R relationship for PM_2.5_. Details of the GEOS-Chem settings and S/R analysis are described in the Methods section.

The model results were analyzed to obtain S/R values. We confirmed that the annual average contribution of Japanese domestic emissions to Fukuoka PM_2.5_ was approximately 20%, and the Chinese contribution was approximately 60% based on the meteorological conditions in 2014^26^. S/R results are very useful for evaluating possible strategies for improving PM_2.5_ levels over downwind regions after enacting appropriate emission controls in one region. For example, the contribution of PM_2.5_ from China (mainly from northern China) to Fukuoka was approximately 60%, and thus if the PM_2.5_ concentration in China decreases by 40% (e.g., from 100 to 60 µg/m^3^ in Beijing between 2014 and 2017), the decrease in PM_2.5_ concentration in Fukuoka can be calculated as follows: 60% × 40% = 24% (assuming that all emissions except those from China remain constant). The observed decrease in PM_2.5_ concentration in Fukuoka (18.5 to 14.5 µg/m^3^) was 22%, which is in good agreement with the model-based S/R estimate.

The contour lines in Fig. 3 represent S/R responses and were calculated by multiplying the fraction of the PM_2.5_ contribution from China at each point by the rate of decrease in Chinese PM_2.5_ concentration. The contours in Fig. 3(a–c) represent rates of decrease over East Asia in response to different decreases in PM_2.5_ concentration in China. The rate of decrease in PM_2.5_ concentration in China was set to 12%, 19%, or 26% based on observations. The observed relationship between rates of decrease in China and Japan can be explained using these contour lines.

For Korea, trends at the upwind background sites of Baengyeongdo and Jeju showed a consistent decreasing signal, in agreement with the decrease based on the S/R relationship. However, trends at other Korean sites cannot be explained by the S/R contour lines, and some cities (e.g., Seoul) exhibited significant increases in PM_2.5_ concentrations in 2016 and 2017 compared with 2015 and large year-to-year variations.

OMI SO_2_ and NO_2_ variations across Korea cannot explain the observed changes in the PM_2.5_ concentration. In Korea, the SO_2_ VCD exhibited a small decrease or no change after 2010, and the NO_2_ VCD remained almost unchanged (see Supplementary Figure S4); thus, the trend of the average PM_2.5_ was not clearly correlated with the local emissions pattern (particularly in urban areas). Elucidating these patterns and their drivers in Korea is a subject for future research.

We examined recent PM_2.5_ and aerosol composition changes over a clean and remote island, Fukue Island, which is located at the western edge of the Japanese mainland and eastern edge of the East China Sea (see Supplementary Figure S1). Details of the observations from Fukue Island and comparison with the GEOS-Chem simulation can be found in the Methods section.

**Figure 4:**
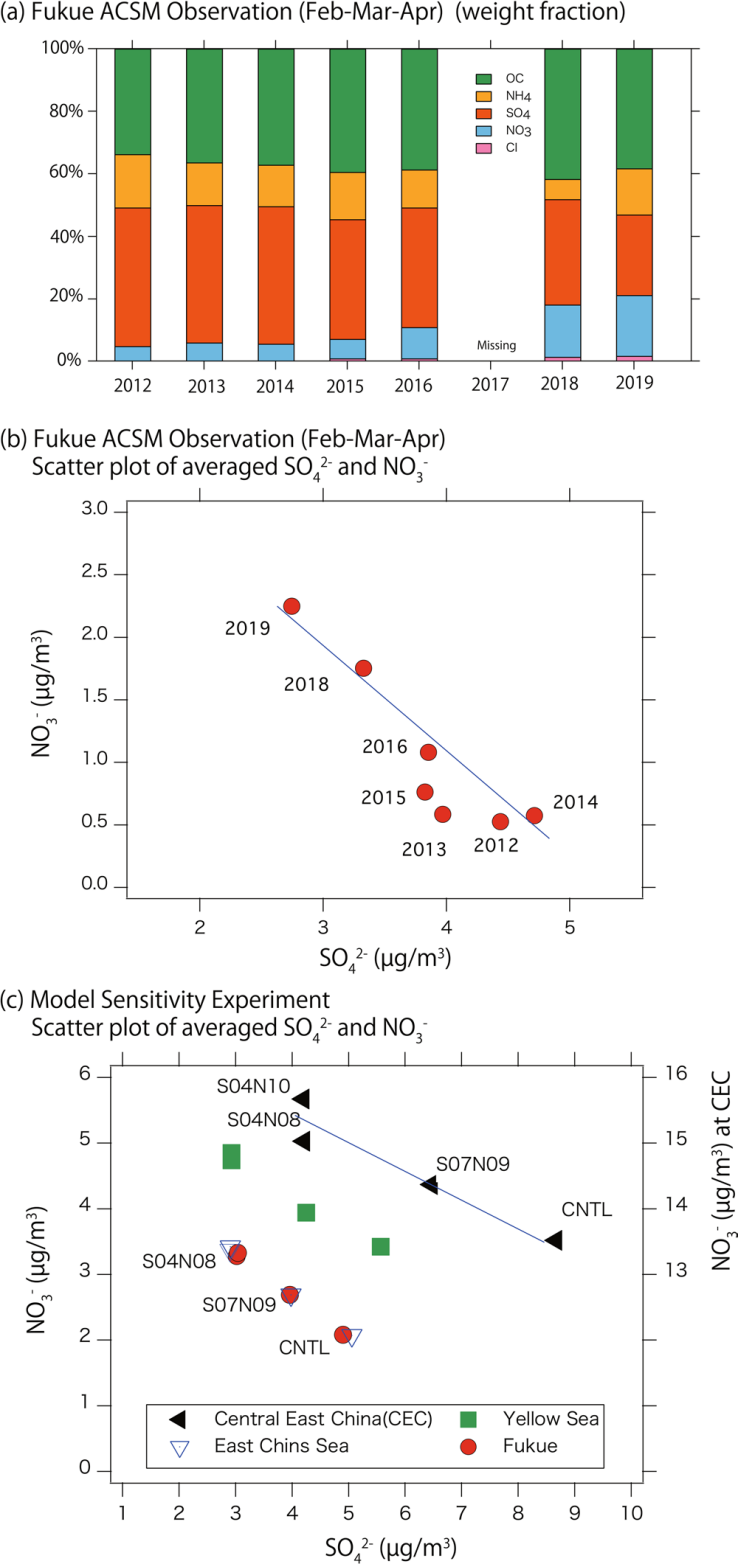
(**a**) Year-to-year changes in aerosol composition observed at Fukue Island (averaged from February to April), (**b**) scatter plot of averaged SO_4_^2−^ and NO_3_^−^ concentrations at Fukue Island from observation data and (**c**) GEOS Chem sensitivity analysis (extracted for CEC, Yellow Sea, East China Sea, and Fukue Island). Model results were averaged from February to April.

Figure 4 shows observation results from Fukue Island averaged between February and April. Figure 4(a) shows the aerosol composition ratios among chloride, NO_3_^−^, SO_4_^2−^, NH_4_^+^, and organic aerosols. Figure 4(b) shows a scatter diagram of SO_4_^2−^ and NO_3_^−^ for each year.

As shown in Fig. 4(a), the observed sulfate concentration decreased significantly (by 40%) at Fukue Island. This result is consistent with the decrease in SO_2_ emissions over China. Although NO_x_ and NH_3_ emissions were also reduced, the observed nitrate concentrations increased continuously. This result could be explained by the chemical balance of the ammonia–nitric acid–sulfuric acid–water system. This thermodynamic equilibrium process is included in the GEOS-Chem simulation described in the Methods section, which allows for detailed studies on chemical balance. Due to the extremely low vapor pressure of sulfuric acid, sulfuric acid produced in the atmosphere consumes ammonia and is neutralized, forming ammonium sulfate aerosol. Then, the leftover ammonia, referred to as free ammonia, is available for the potential formation of ammonium nitrate. As a result, the reduction of sulfuric acid causes more free ammonia to be available, leading to the formation of more ammonium nitrate. Seinfeld and Pandis (2016)^27^ indicates that about half of the decrease in concentration of (NH_4_)_2_SO_4_ will be offset by the increase in NH_4_NO_3_. The relationship between the decrease in sulfate and increase in nitrate depends primarily on the concentrations of their precursors, relative humidity (RH), and temperature.

Although SO_2_, NO_x_, and NH_3_ emissions over China have all been reduced, the decrease in NO_x_ is significantly smaller than that in SO_2_, and the decrease in NH_3_ is much smaller than that in either SO_2_ or NO_x_ (see Fig. 1). If the increase in nitrate due to SO_2_ reduction is larger than the nitrate decreases due to decreases in NO_x_ and NH_3_ emissions, the overall effects of emission control will lead to increased nitrate concentration. These phenomena were actually observed on Fukue Island from 2012 to 2019 (as seen in Fig. 4b), where sulfate decreased by 1.7 μg/m^3^, while nitrate increased by 1.7 μg/m^3^, causing the NO_3_^−^ concentration to increase by almost four-fold compared to the 2012–2014 period. A more detailed analysis of these phenomena based on the GEOS-Chem model is described below.

## Discussion

To quantitatively analyze the increase in NO_3_^−^, we modeled an additional four cases of sensitivity experiments, changing the SO_2_ and NO_x_ emission intensities based on the bottom-up Multi-resolution Emission Inventory for China (MEIC)^1^ results (Table 1). Emission reduction was applied only in the China region, and emissions in all other regions were the same as in the control experiment.

**Table 1:** Design of a model for sensitivity analysis of Chinese emissions.

Case Code	Purpose	SO_2_ emission	NO_x_ emission
CNTL (S10N10)	Control experiment	100%	100%
S04N08	Decrease relative to Fig. 1	40%	80%
S07N09	SO_2_ linearity	70%	90%
S04N10	NO_x_ sensitivity	40%	100%

CNTL (S10N10) was the control experiment. Case S04N08 was designed based on the MEIC emission reduction rate and OMI satellite changes, and thus is suitable for examining recent emission changes. Case S07N09 was designed to examine the linearity of decreases in SO_2_ (between the S10, S07, and S04 cases), and case S04N10 was designed to examine NO_x_ sensitivity under a constant SO_2_ condition (with S04N08). In this sensitivity study, emissions of NH_3_ and non-methane volatile organic compounds were the same as in CNTL.

The results of the modeled sensitivity experiments are shown in Fig. 4(c) for SO_4_^2−^ and NO_3_^−^ in CEC, the centers of the Yellow Sea and East China Sea, and Fukue Island (model results were averaged between February and April for consistency with observations). Note that Fig. 4(c) demonstrates the typical response of the ammonia–nitric acid–sulfuric acid–water system to the emission sensitivity shown in Table 1. Thus, the absolute concentration level is different from the ACSM observation, but the fundamental changes observed can be explained by the model emission sensitivity experiment.

As shown in Fig. 4(c), the sensitivity experiment between CNTL (=S10N10) and S04N10 for CEC showed decreased SO_4_^2−^ (ΔSO_4_^2−^ = −4.5 µg/m^3^) and increased NO_3_^−^ (ΔNO_3_^−^ = +2.2 µg/m^3^), consistent with the response discussed in the Results section. The series of sensitivity experiments (CNTL, S07N09 and S04N08) shows a nearly linear response between ΔSO_4_^2−^ and ΔNO_3_^−^ even with decreasing NO_x_ emission. The differing responses of NO_3_^−^ in S04N08 and S04N10 (NO_x_ emission difference) is most apparent for Beijing. This difference becomes very small over the downwind regions of the East China Sea and Fukue Island. The ratio of ΔSO_4_^2−^: ΔNO_3_^−^ ranges from 1:0.5 to 1:0.65, becoming larger as the transport distance increases. As noted above, the relationship between the decrease of sulfate and the increase of nitrate is strongly dependent on RH, temperature and a heterogeneous reaction with sea salt (for NaNO_3_ formation) during transport from mainland China over the ocean. These responses are quite consistent with observations at Fukue Island.

The change in NH_3_ concentration between CNTL and S04N08 is of great interest, and this result is shown in Supplementary Figure S5. The NH_3_ concentration in the S04N08 experiment was more than double that over CEC (i.e., increase in free NH_3_)^28^, and NH_3_ concentration increases were simulated over western Japan, including Fukue Island. The changes in NH_3_ concentration over CEC were also supported by Infrared Atmospheric Sounding Interferometer (IASI) satellite observations^29^. The conclusions of these sensitivity studies were reasonable, showing that reductions in SO_2_ emissions change the balance of the ammonia–nitric acid–sulfuric acid–water system, creating free NH_3_ that reacts with HNO_3_ to form NH_4_NO_3_, which is transported to downwind regions, especially in the cold season.

**Figure 5:**
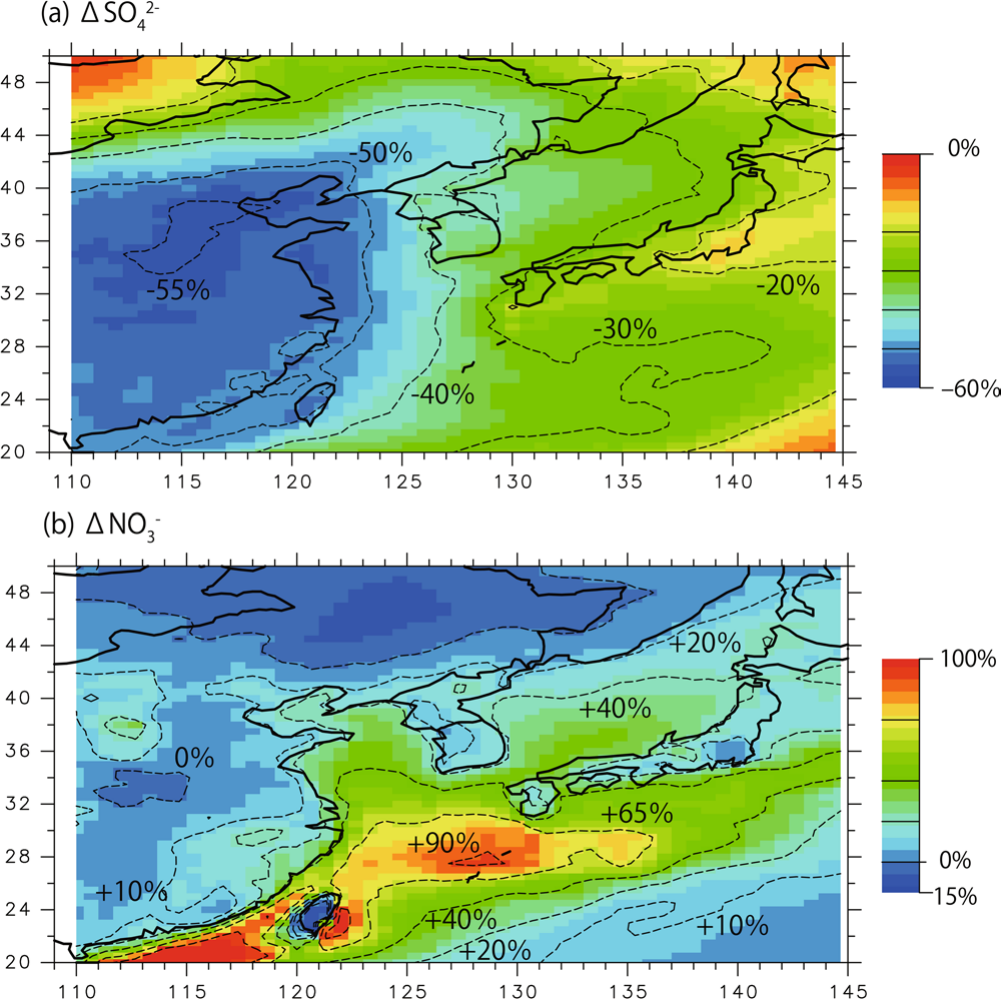
Horizontal distribution of relative changes in (**a**) SO_4_^2−^ and (**b**) NO_3_^−^ concentrations between the S04N08 sensitivity and CNTL cases based on annually averaged concentrations (see Eq. 2 for definitions).

Figure 5 shows the horizontal distribution of scaled annual mean ΔSO_4_^2−^ and ΔNO_3_^−^ from the model sensitivity study, based on the CNTL and S04N08 experiments. These indices are calculated as follows:

2a $${{\Delta {\rm{S}}{\rm{O}}}_{4}}^{2-}=({{\rm{S}}{\rm{O}}}_{4}^{2-}{}_{{}_{-}{\rm{S}}04{\rm{N}}08}{\text{-}{\rm{S}}{\rm{O}}}_{4}^{2-}{}_{{}_{-}{\rm{C}}{\rm{N}}{\rm{T}}{\rm{L}}}){/{\rm{S}}{\rm{O}}}_{4}^{2-}{}_{{}_{-}{\rm{C}}{\rm{N}}{\rm{T}}{\rm{L}}}$$

2b $${{\Delta {\rm{N}}{\rm{O}}}_{3}}^{-}=({{\rm{N}}{\rm{O}}}_{3}^{-}{}_{{}_{-}{\rm{S}}04{\rm{N}}08}{\text{-}{\rm{N}}{\rm{O}}}_{3}^{-}{}_{{}_{-}{\rm{C}}{\rm{N}}{\rm{T}}{\rm{L}}}){/{\rm{N}}{\rm{O}}}_{3}^{-}{}_{{}_{-}{\rm{C}}{\rm{N}}{\rm{T}}{\rm{L}}}$$

The SO_4_^2−^ decrease (ΔSO_4_^2−^) over mainland China exceeded −50%, consistent with the 60% decrease in SO_2_ (Fig. 1a). Over western and eastern Japan, SO_4_^2−^ decreased by 30% and 20%, respectively. We found that this rate of decrease was linearly proportional to the SO_2_ reduction rate within China via a comparison with case S07N09. The impacts of the decrease in SO_2_ in China clearly covered a large area downwind.

For NO_3_^−^, ΔNO_3_^−^ over China was not significant, which is consistent with recent observations^9^. Several areas downwind of China exhibited increased NO_3_^−^. Over the East China Sea, the rate of increase in NO_3_^−^ exceeded 90%, as NO_3_^−^ concentrations were low in this area in the CNTL experiment; thus, a small increase in NO_3_^−^ results in a large rate of increase. In the Fukue Island region, this increase was approximately 60%. Figure 5 shows annually averaged values, and ratios increased when averaged over the cold season (February to April) because NH_4_NO_3_ is more stable in cold weather, as discussed below.

**Figure 6:**
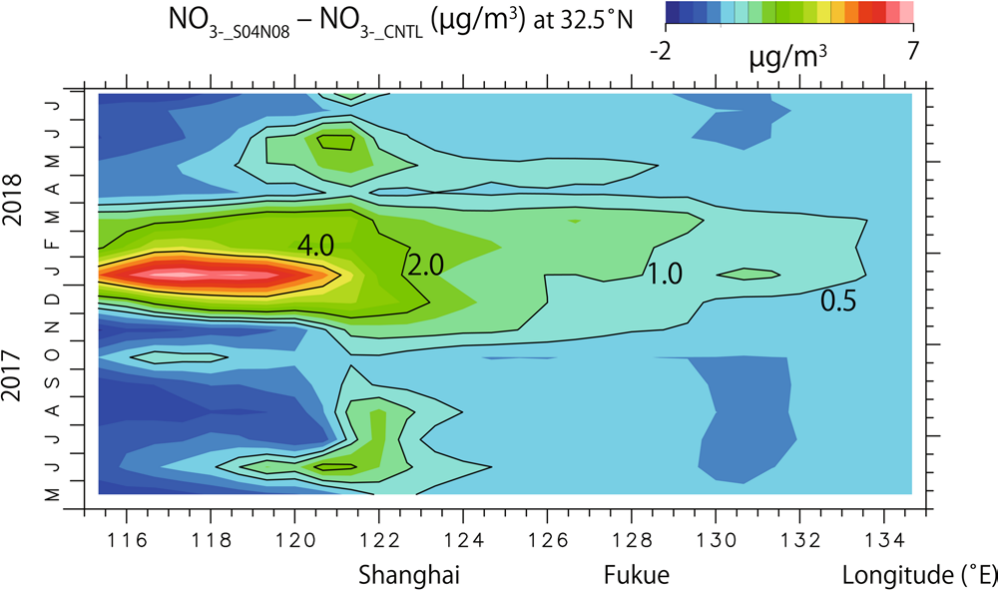
Time-longitude plot of the absolute concentration differences in NO_3_^−^ (values of S04N08 sensitivity experiment – those of CNTL case) between March 2017 and July 2018 along the 32.5° N latitude line (model vertical level = 1).

Figure 6 shows time–longitude trends of the NO_3_^−^ increase from experiments CNTL to S04N08 along the latitude of 32.5° N. This latitude corresponds the typical transport route from Shanghai to Fukue Island. The increase in concentrations over the downwind regions of China between December and March was significant and was caused by cold weather increasing NH_4_NO_3_ stability. During the warm season, the transport path changes and warm temperatures cause NH_4_NO_3_ aerosols to enter the gas phase as HNO_3_. At Fukue Island, the increase reached 1 µg/m^3^ in winter, consistent in magnitude with observations at Fukue Island. Notably, the eastern edge of area in which NO_3_^−^ increased (on the order of 0.5 µg/m^3^) approaches 134° E to 136° E, where large cities such as Osaka are located. Note that the changes in concentration were small and usually difficult to detect from observations over urban areas of mainland Japan due to large local NO_x_ emissions. However, this small increase may contribute significantly to the presence of excess nitrogen over the downwind region in East Asia^23^.

Recent studies have described how a decrease in PM_2.5_ can enhance the lifetime of OH radicals and increase the O_3_ level^30^ (followed by increases in the atmospheric oxidation capacity and NO_3_^−^ formation). This is a reasonable mechanism that might increase the NO_3_^−^ formation. However, our version of the GEOS-Chem model does not include the heterogeneous interaction between PM_2.5_ and OH. Our results explain the observed SO_4_^2−^/NO_3_^−^ changes exactly, and this indicates that a change in the atmospheric oxidation capacity is not the primary reason for the observed changes in SO_4_^2−^/NO_3_^−^.

We analyzed the PM_2.5_ observation data from 2014 to 2019 over Japan, Korea, and China, and found that there was a clear decreasing trend over Japan, which was strongly correlated with levels in China. An emission sensitivity study based on the GEOS-Chem chemical transport model was carried out to quantify the relationship between emission levels in China and PM_2.5_ concentrations over downwind regions. The model results showed that the trend of an annual decrease in PM_2.5_ in Japan was explained primarily by reduced PM_2.5_ concentrations in China. We also used this model to quantitatively evaluate the impact of Chinese environmental improvements on downwind areas using S/R analysis. Rapid emission reductions played an important role in reducing PM_2.5_ concentrations, but a chemical imbalance in the ammonia–nitric acid–sulfuric acid–water system caused an increase in long-range NO_3_^−^ transport to downwind regions. Observations on a clean remote island and numerical modeling confirmed that this paradigm shift has occurred since 2014–2015. Concentrations of sulfate, a chemical that undergoes long-range transport, are decreasing, whereas those of nitrate, which is subject only to short-distance transport, are increasing. This increase in nitrate could lead to an excess nitrogen burden in East Asia and the surrounding oceanic regions^31,32^. We found that the most recent satellite NO_2_ and SO_2_ VCDs, for 2019 (see Supplementary Figure S4), revealed that this paradigm shift is accelerating (because SO_2_ is still decreasing, whereas NO_2_ is now increasing), indicating that there is a need for careful continuous observation of changes in aerosol chemical compositions, both in China and the downwind regions of Japan and Korea.

## Methods

### Surface PM_2.5_ observation data

For Japan, hourly PM_2.5_ observation data from the Atmospheric Environmental Regional Observation System^33^ (AEROS; also referred to as ‘Soramame-kun’) were used for calculating annually averaged PM_2.5_ concentrations from 2013 to 2018. A total of 662 AEROS sites were used in this study, and after quality control processing, the AEROS data were interpolated into a 0.375° longitude-latitude grid. Data averaged across Japan were obtained from the Ministry of Environment of Japan.

For China, PM_2.5_ concentration data were obtained from the China National Environmental Monitoring Center^34^. In China, PM_2.5_ concentrations have been monitored in 74 major cities since the end of 2012, including cities in the Beijing-Tianjin-Hebei region, Yangtze River Delta, and Pearl River Delta, as well as Chongqing municipalities and all provincial capitals. Data from these 74 cities were collected between 2013 and 2018, averaged, and used for analysis in this study. We also analyzed PM_2.5_ observations taken at the U.S. Embassy in Beijing from 2011 to 2019.

For Korea, the annual average PM_2.5_ values were obtained from the official Air Korea website of the Ministry of Environment of Korea^35^. We selected nine sites, including the cities of Seoul, Busan, Gwangju, Gangneung, Deagu, Daejeon and Mokpo, as well as Jeju and the background site of Baengnyeongdo (the locations of the latter two sites are shown in Supplementary Figure S1) for analysis between 2015 and 2018. Data collected from multiple points within large cities were averaged.

We used observation sites with more than 250 days of qualified observations.

### Chemical transport model and S/R analysis

We used the GEOS-Chem model for analysis^25^. The model was run using the full GEOS-Chem NOx-Ox-VOC-HOx-CO chemistry option to simulate the formation of aerosols, including mineral dust, sea salt, black carbon (BC), organic carbon (OC), and secondary inorganic aerosols. The GEOS-Chem model used ISORROPIA-II^36^ to calculate the detailed thermodynamic equilibrium processes for the H^+^–NH_4_^+^–K^+^–Ca^2+^–Mg^2+^–Na^+^–OH ^−^–SO_4_^2−^–NO_3_^−^–Cl^−^–H_2_O aerosol system. The model used the assimilated meteorological fields from GEOS of the NASA Global Modeling and Assimilation Office. The model has a horizontal resolution of 2° × 2.5° for global runs, and 0.5° × 0.667° for Asian one-way nesting runs (11° S−55° N, 70−150° E), both containing 47 vertical levels from the surface to 0.01 hPa. We used anthropogenic emissions data from the Emission Database for Global Atmospheric Research^37^ for the global domain and from the Regional Emission Inventory in Asia (REAS; ver. 2.1) for the Asian domain^38^. REAS NH_3_ emissions data were modified to include seasonal variations in China^39^. PM_2.5_ concentrations from the model were calculated by summing the concentrations of relevant aerosols (BC, OC, SO_4_^2−^, NO_3_^−^, NH_4_^+^, dust, and sea salt). Model simulation was conducted from the beginning of December 2013 to the end of July 2019, and the results from the first 8 months were used for model training. We primarily used the S/R model results for 2014 (when the pollution level was high) and assumed that the model results would be similar for the meteorology of different years. Other basic numerical settings were as reported in Uno *et al*. ^18^. We set 19 source regions (including Japan, Korea, northern China, and central China) for S/R analysis^26^. We used a 20% reduction of emission (not zero emission) to avoid undesirable non-linearity of the chemical reactions. The source contribution from region A is calculated as follows:

$${\rm{Source}}\,{\rm{contribution}}\,{\rm{from}}\,{\rm{region}}\,{\rm{A}}=({{\rm{PM}}}_{100,{\rm{all}}}-{{\rm{PM}}}_{80,{\rm{A}}})/({{\rm{PM}}}_{100,{\rm{all}}}-{{\rm{PM}}}_{80,{\rm{all}}}),$$

where PM_100,all_ is the PM_2.5_ concentration under the 100% emission scenario for all regions.

### Aerosol observations using the ACSM at Fukue Island

The chemical compositions and mass concentrations of atmospheric fine aerosols, i.e., fine particulate matter (PM_1_), were observed at the remote island of Fukue, Nagasaki Prefecture, Japan (32.75° N, 128.68° E; see Supplementary Figure S1). The population on this island is approximately 40,000 and it is generally considered to have few emission sources. Aerosol chemical composition was measured using a quadrupole-type ACSM (Q-ACSM; Aerodyne Research Inc., Billerica, MA, USA). The mass concentrations of PM_2.5_ were obtained from an air-pollution monitoring station at Goto (located on Fukue Island), which is the site of municipal government offices for Nagasaki prefecture. The chemical compositions of ammonium, nitrate, sulfate, chloride, and organic compounds were analyzed. Because our main interest was trans-boundary air pollution from mainland China, measurements were taken only from January to May on Fukue Island. The details of Q-ACSM and calibration procedures for Fukue Island have been described previously^22,40,41^. To confirm the model simulation, the ACSM observations and GEOS-Chem model results were compared over a 4-month period (Supplementary Figure S6). The GEOS-Chem experiment (CNTL) used emissions from 2010, and thus SO_4_^2−^ and NO_3_^−^ concentrations from GOES-Chem were over- and underestimated, respectively, but we confirmed that the model results reproduce the observed variations well.

## Supplementary information

Supplementary information.

## Data Availability

The datasets generated for the present study are available from the corresponding authors upon reasonable request.
