# Supplementary material for: Paradigm shift in aerosol chemical composition over regions downwind of China
Source: Sci Rep. 2020 Apr 15;10:6450. doi: 10.1038/s41598-020-63592-6 (PMC7160133; doi:10.1038/s41598-020-63592-6)
Supplement: Supplementary file 1 — Supplementary information. [file 41598_2020_63592_MOESM1_ESM.pptx]

## Slide 1
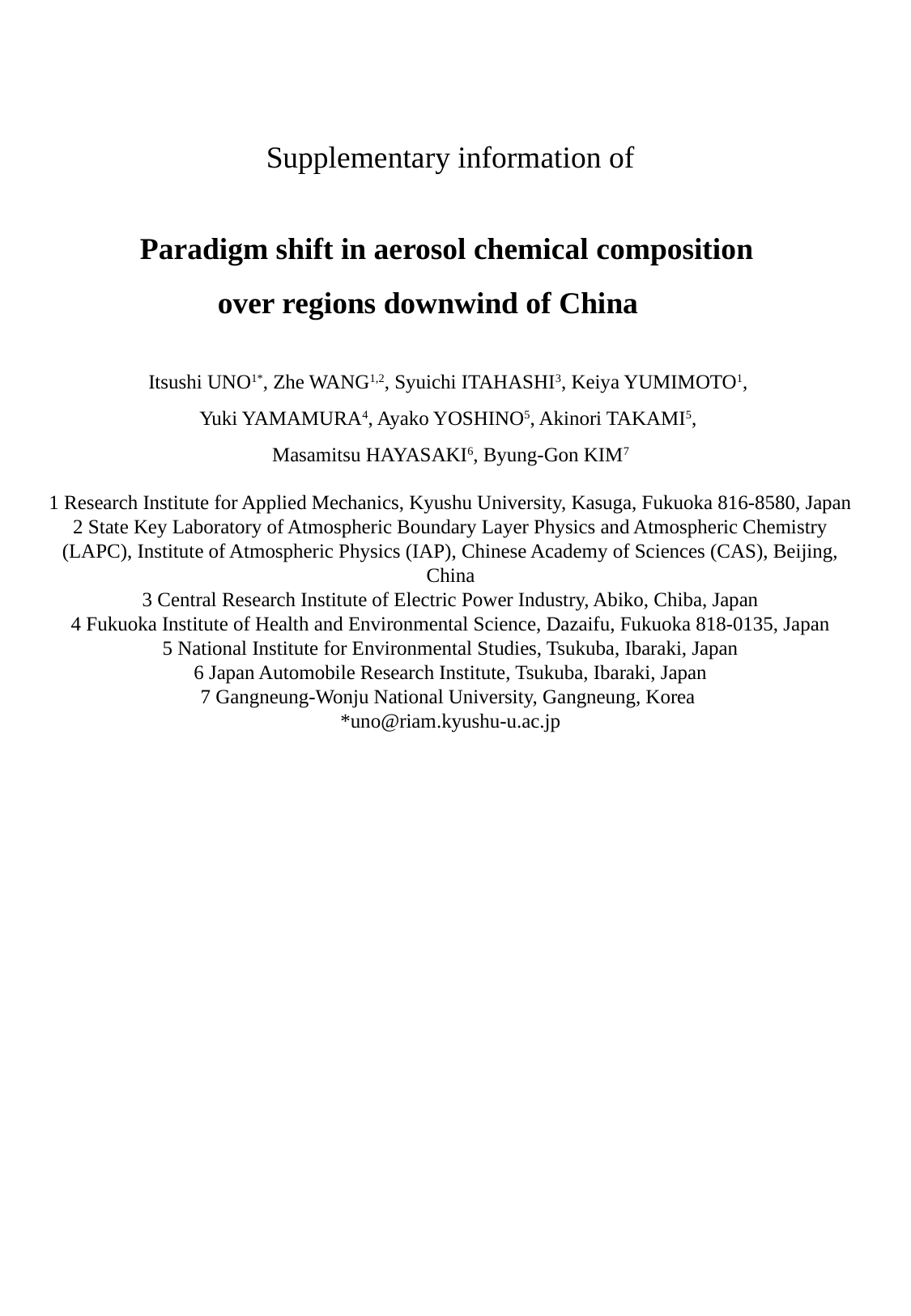

Supplementary information of
Paradigm shift in aerosol chemical composition
over regions downwind of China
Itsushi UNO1*, Zhe WANG1,2, Syuichi ITAHASHI3, Keiya YUMIMOTO1,
Yuki YAMAMURA4, Ayako YOSHINO5, Akinori TAKAMI5,
Masamitsu HAYASAKI6, Byung-Gon KIM7
1 Research Institute for Applied Mechanics, Kyushu University, Kasuga, Fukuoka 816-8580, Japan
2 State Key Laboratory of Atmospheric Boundary Layer Physics and Atmospheric Chemistry (LAPC), Institute of Atmospheric Physics (IAP), Chinese Academy of Sciences (CAS), Beijing, China
3 Central Research Institute of Electric Power Industry, Abiko, Chiba, Japan
4 Fukuoka Institute of Health and Environmental Science, Dazaifu, Fukuoka 818-0135, Japan
5 National Institute for Environmental Studies, Tsukuba, Ibaraki, Japan
6 Japan Automobile Research Institute, Tsukuba, Ibaraki, Japan
7 Gangneung-Wonju National University, Gangneung, Korea
*uno@riam.kyushu-u.ac.jp

## Slide 2
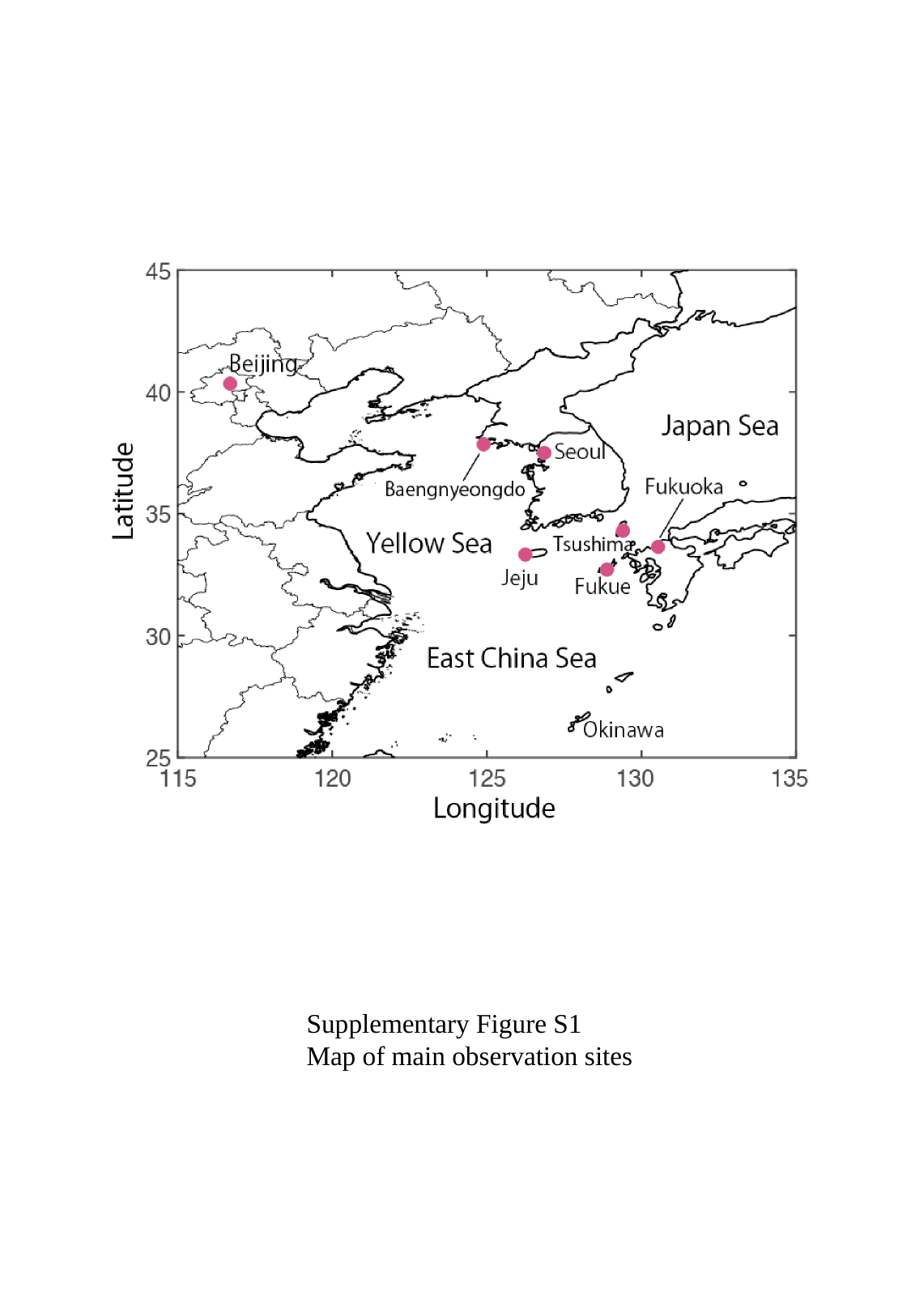

Supplementary Figure S1
Map of main observation sites

## Slide 3
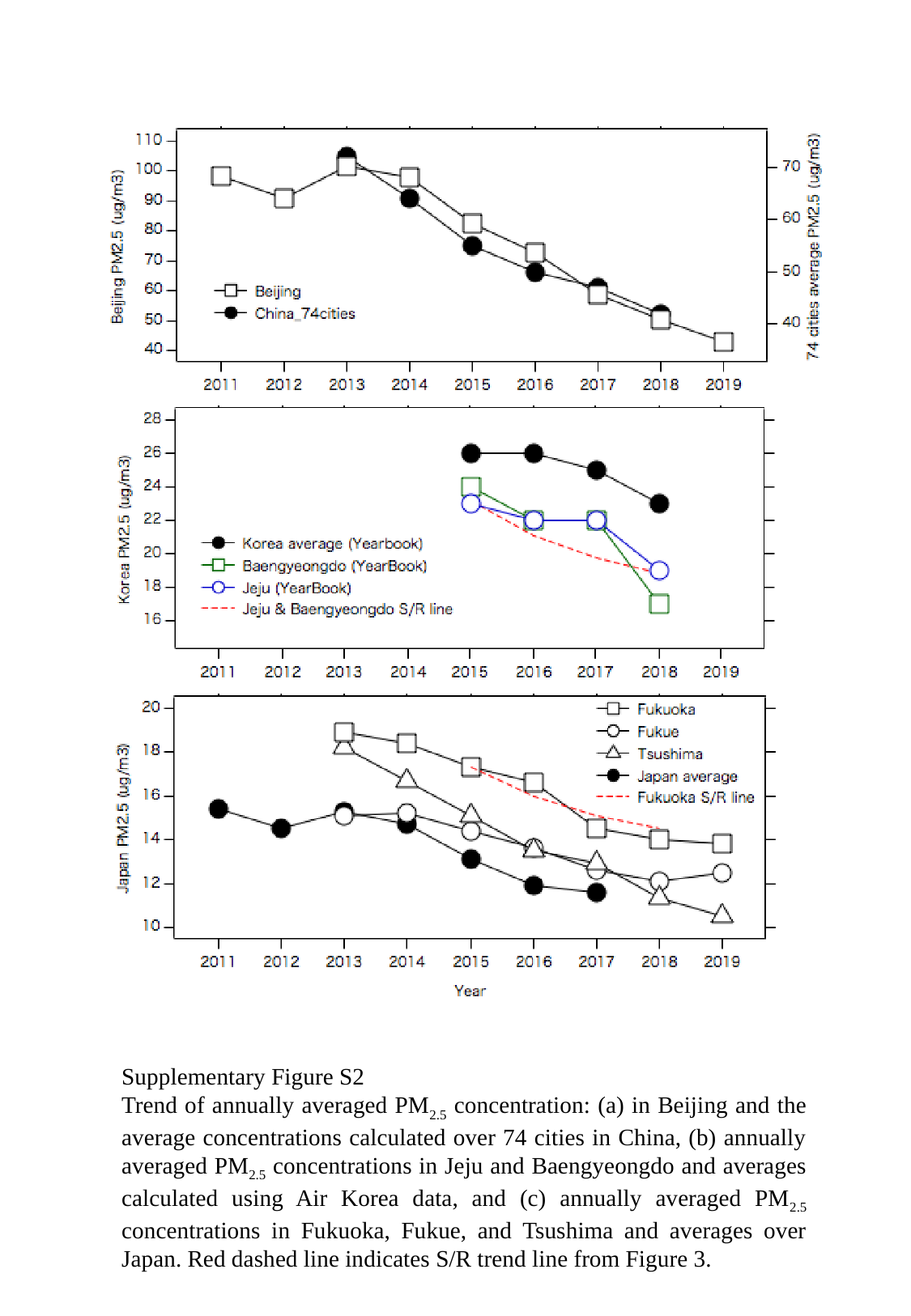

Supplementary Figure S2
Trend of annually averaged PM2.5 concentration: (a) in Beijing and the average concentrations calculated over 74 cities in China, (b) annually averaged PM2.5 concentrations in Jeju and Baengyeongdo and averages calculated using Air Korea data, and (c) annually averaged PM2.5 concentrations in Fukuoka, Fukue, and Tsushima and averages over Japan. Red dashed line indicates S/R trend line from Figure 3.

## Slide 4
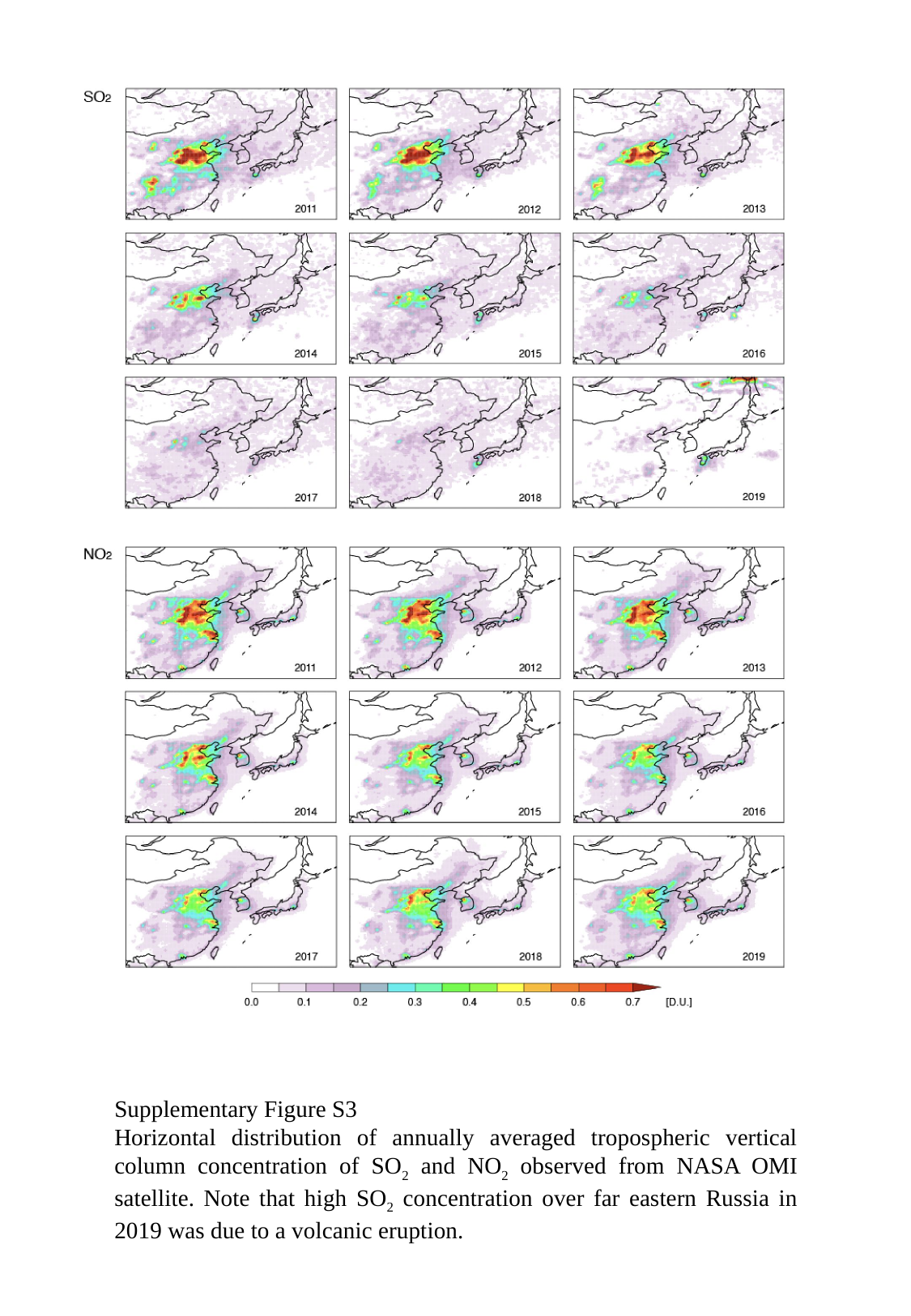

Supplementary Figure S3
Horizontal distribution of annually averaged tropospheric vertical column concentration of SO2 and NO2 observed from NASA OMI satellite. Note that high SO2 concentration over far eastern Russia in 2019 was due to a volcanic eruption.

## Slide 5
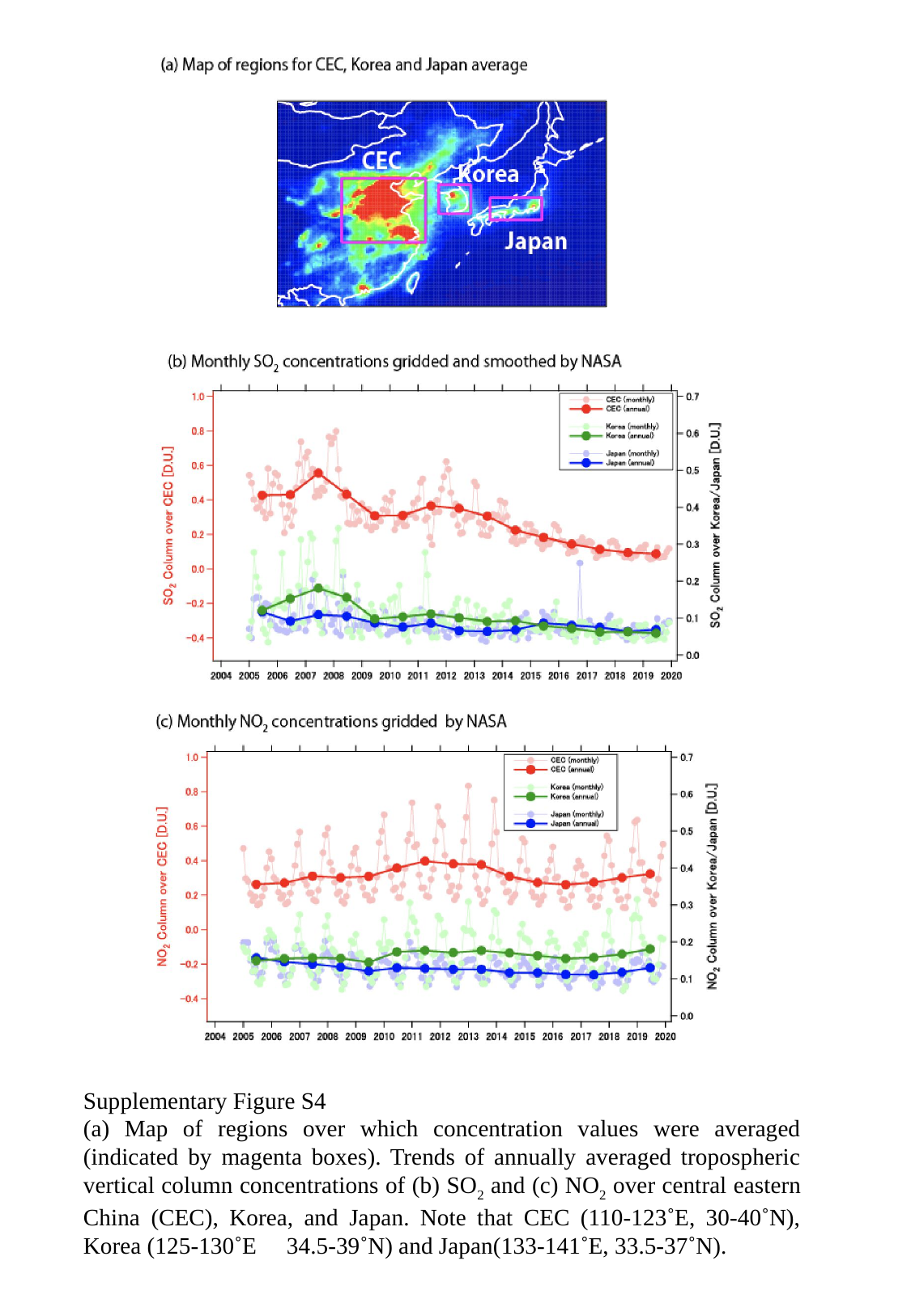

Supplementary Figure S4
(a) Map of regions over which concentration values were averaged (indicated by magenta boxes). Trends of annually averaged tropospheric vertical column concentrations of (b) SO2 and (c) NO2 over central eastern China (CEC), Korea, and Japan. Note that CEC (110-123˚E, 30-40˚N), Korea (125-130˚E，34.5-39˚N) and Japan(133-141˚E, 33.5-37˚N).

## Slide 6
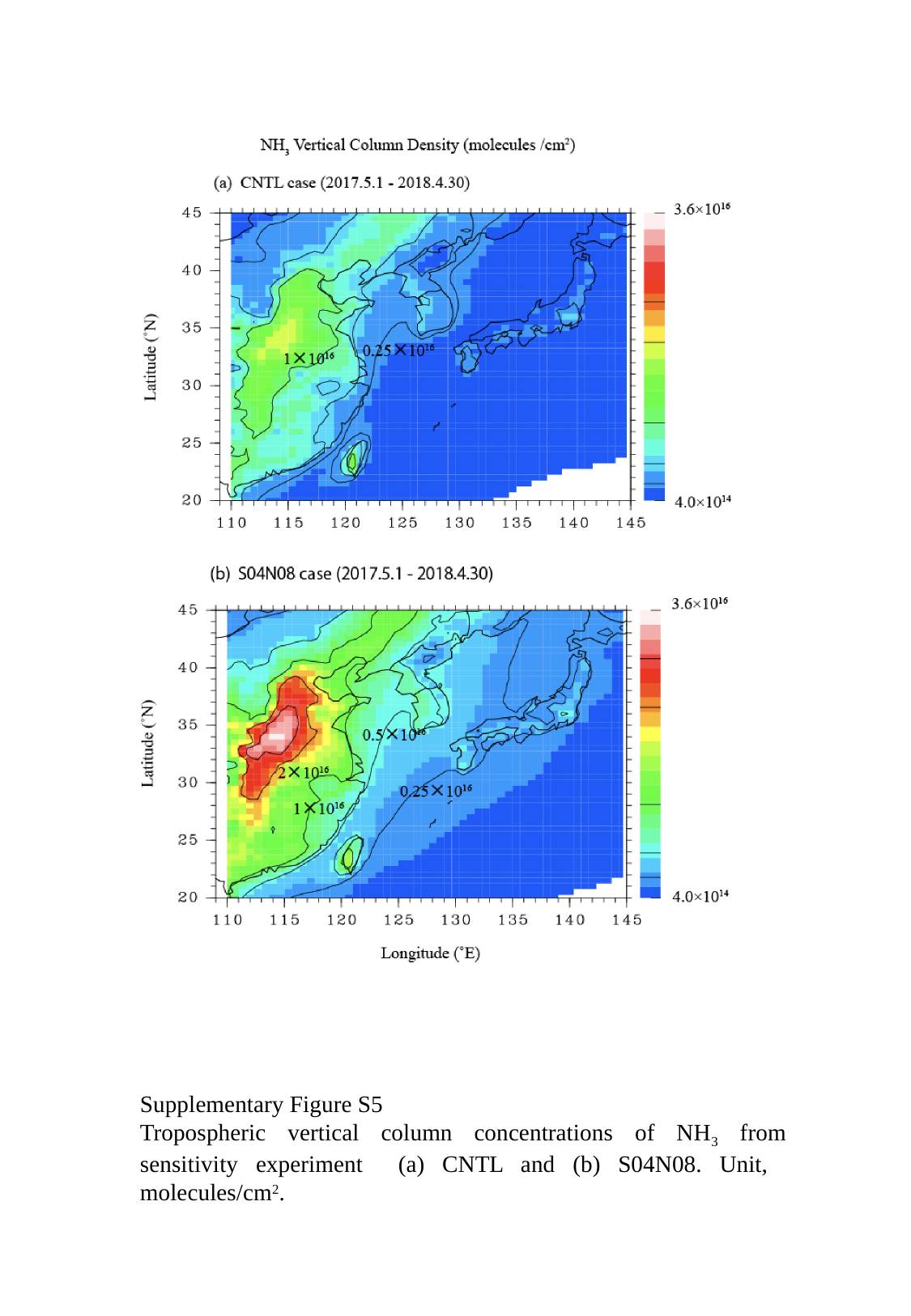

Supplementary Figure S5
Tropospheric vertical column concentrations of NH3 from sensitivity experiment (a) CNTL and (b) S04N08. Unit, molecules/cm2.

## Slide 7
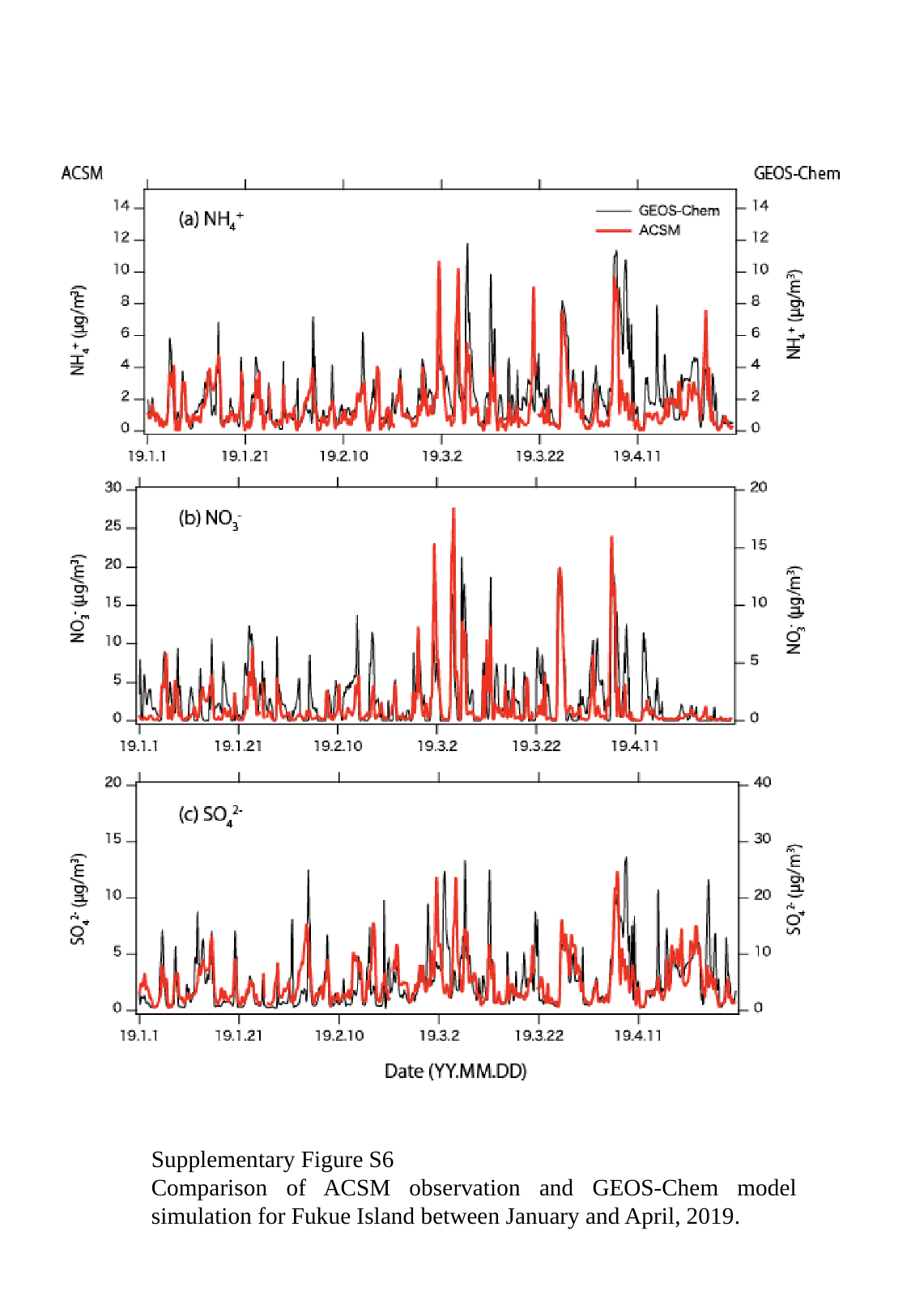

Supplementary Figure S6
Comparison of ACSM observation and GEOS-Chem model simulation for Fukue Island between January and April, 2019.
